# Trastuzumab-based treatment of HER2-positive breast cancer: an antibody-dependent cellular cytotoxicity mechanism?

**DOI:** 10.1038/sj.bjc.6602930

**Published:** 2006-01-10

**Authors:** L Arnould, M Gelly, F Penault-Llorca, L Benoit, F Bonnetain, C Migeon, V Cabaret, V Fermeaux, P Bertheau, J Garnier, J-F Jeannin, B Coudert

**Affiliations:** 1 Department of Pathology, Centre G-F Leclerc, Dijon 21000, France; 2 Centre J Perrin, Clermont 63011, France; 3 Department of Surgery, Centre G-F Leclerc, Dijon 21000, France; 4 Department of Statistics Centre G-F Leclerc, Dijon 21000, France; 5 Centre A Vautrin, Nancy 54000, France; 6 Centre O Lambret, Lille 59020, France; 7 CHU, Limoge 87000, France; 8 CHU Saint Louis, Paris 75010, France; 9 Laboratoire Roche, Neuilly 92521, France; 10 EPHE, INSERM U517, and IFR 100, Faculté de médecine, Dijon 21063, France; 11 Department of Oncology, Centre G-F Leclerc, Dijon 21000, France

**Keywords:** primary systemic therapy, trastuzumab, ADCC, MoA

## Abstract

This study evaluated by immunohistochemistry (IHC) immune cell response during neoadjuvant primary systemic therapy (PST) with trastuzumab in patients with HER2-positive primary breast cancer. In all, 23 patients with IHC 3+ primary breast cancer were treated with trastuzumab plus docetaxel. Pathological complete and partial responses were documented for nine (39%) and 14 (61%) patients, respectively. Case-matched controls comprised patients treated with docetaxel-based PST without trastuzumab (D; *n*=*23*) or PST without docetaxel or trastuzumab (non-taxane, non-trastuzumab, NT–NT; *n*=*23*). All surgical specimens were blind-analysed by two independent pathologists, with immunohistochemical evaluation of B and T lymphocytes, macrophages, dendritic cells and natural killer (NK) cells. Potential cytolytic cells were stained for Granzyme B and TiA1. HER2 expression was also evaluated in residual tumour cells. Trastuzumab treatment was associated with significantly increased numbers of tumour-associated NK cells and increased lymphocyte expression of Granzyme B and TiA1 compared with controls. This study supports an *in vivo* role for immune (particularly NK cell) responses in the mechanism of trastuzumab action in breast cancer. These results suggest that trastuzumab plus taxanes lead to enhanced NK cell activity, which may partially account for the synergistic activity of trastuzumab and docetaxel in breast cancer.

The human epidermal growth factor receptor 2 (HER2) is a member of the ErbB family that plays an important role in promoting oncogenic transformation and tumour growth (Slamon *et al*, 1987). Approximately 25–30% of patients with breast cancer overexpress HER2 and this overexpression is correlated with gene amplification and poor clinical outcome (King *et al*, 1985; Slamon *et al*, 1987, 1989; van de Vijver *et al*, 1988; Gusterson *et al*, 1992; Hynes and Stern, 1994).

Trastuzumab (Herceptin®; F Hoffmann-La Roche, Basel, Switzerland) selectively targets HER2 and is approved for the treatment of women with HER2-overexpressing metastatic breast cancer (MBC). Trastuzumab demonstrates favourable efficacy both as a single agent (Cobleigh *et al*, 1999; Vogel *et al*, 2002) and in combination with cytotoxic chemotherapy (Pegram *et al*, 1999; Slamon *et al*, 2001; Marty *et al*, 2005). The proposed mechanisms of action of trastuzumab include enhancement of HER2 degradation (Molina *et al*, 2001), inhibition of cell cycle progression via inhibition of the mitogen-activated protein kinase pathway (Le *et al*, 2003; Jackson *et al*, 2004), and suppression of the antiapoptotic phosphatidylinositol 3-kinase and Akt pathways (Yakes *et al*, 2002; Mohsin *et al*, 2005). In addition, there is evidence supporting a role for trastuzumab in mediating antibody-dependent cellular cytotoxicity (ADCC) (Cooley *et al*, 1999; Clynes *et al*, 2000; Carson *et al*, 2001; Repka *et al*, 2003; Gennari *et al*, 2004). Mechanisms of action of trastuzumab demonstrated *in vitro* in HER2-overexpressing cells are not always confirmed in *in vivo* studies (Mohsin *et al*, 2005).

Trastuzumab-based therapy has been shown to be effective in the neoadjuvant (primary systemic therapy (PST)) setting (Bines *et al*, 2003; Burstein *et al*, 2003; Schiffhauer *et al*, 2003; Van Pelt *et al*, 2003; Baselga *et al*, 2004; Buzdar *et al*, 2005). This immunohistological analysis, which was undertaken as part of a clinical study, aimed to evaluate the immune response to trastuzumab-based PST within tumours obtained from women with locally advanced breast cancer.

## PATIENTS AND METHODS

### Patients

Between March 2001 and December 2003, 33 women (age 18–65 years) with stage II/III, unilateral, non-inflammatory, operable breast cancer requiring a mastectomy (but who wished to conserve the breast) were enrolled in the open-label, multicentre, phase II TAXHER01 trial (Coudert *et al*, 2005). All patients had HER2-positive (immunohistochemistry (IHC) 3+ or fluorescence *in-situ* hybridisation (FISH)-positive) breast cancer and were treated with PST consisting of six cycles of docetaxel (100 mg m^−2^ 60-min intravenous (i.v.) infusion every 3 weeks) and trastuzumab (4 mg kg^−1^ 90-min i.v. infusion 1 day before the first dose of docetaxel, and thereafter at a dose of 2 mg kg^−1^ weekly for 17 weeks). All patients underwent surgery 3 weeks after the last cycle of docetaxel and trastuzumab.

In all, 46 patients with breast cancer enrolled in the GIREC01 trial (Luporsi *et al*, 2000) were used as matched controls. Of these patients, 23 received anthracycline-based, non-taxane, non-trastuzumab-containing PST (NT–NT; six cycles), and 23 received docetaxel-based, non-trastuzumab-containing therapy (D; six cycles). Tumours from 15 patients in the control group were HER2 positive, five in the NT–NT group and 10 in the D group. The treatment group (TAXHER01) was case-matched with the two control groups in terms of pathologic tumour and node response. Due to the lack of patients treated preoperatively with such a combination, it was not possible to have control groups with trastuzumab plus a non-taxane chemotherapy or trastuzumab alone.

Both clinical studies were conducted in accordance with the Helsinki Declaration and approved by an independent ethics committee. Written informed consent was obtained from all patients prior to enrolment.

### Microscopic evaluation of the pathologic response to PST

Microtome sections obtained from surgical tissue were fixed in 10% neutral buffered formaldehyde solution or Bouin fluid (Richard-Allan Scientific, Kalamazoo, MI, USA), embedded in paraffin wax, and stained with haematoxylin, eosin, and saffron. Pathologic response to PST was assessed according to the Sataloff classification (Sataloff *et al*, 1995). Tumour samples containing no tumour nodules or tumours with residual area <2 mm in diameter were classified as TA. Tumour samples with residual tumour area <0.2 mm in diameter in lymph nodes, not classified as micrometastases in the UCC classification (Singletary *et al*, 2002; Singletary and Greene, 2003), were classified as NA.

### Microscopic and immunohistologic analyses

Immunohistochemical evaluation was performed on one paraffin block that was considered to correspond morphologically with the tumour response area, using the biotin–streptavidin complex (ABC) technique (StreptABC complex/HRP Duet; Dako, Glostrup, Denmark) and the following primary antibodies: B lymphocytes (CD20 (L26, 1 out of 50; Dako, Glostrup, Denmark)); T lymphocytes (CD3 (F7.2.38, 1 out of 25; Dako, Glostrup, Denmark); CD4 (NCL-CD4-368, 1 out of 150; Novocastra, Newcastle, UK); CD8 (C8/144B, 1 out of 25; Dako, Glostrup, Denmark)); macrophages (CD68 (PG-M1, 1 out of 50; Dako, Glostrup, Denmark)); dendritic cells (PS100 (S100, 1 out of 500; Dako, Glostrup, Denmark); CD1a (010, 1 out of 40; Dako, Glostrup, Denmark)); HLA-DR-expressing cells (HLA-DR (TAL.1B5, 1 out of 20;Dako, Glostrup, Denmark)) and natural killer (NK) cells (CD56 (1B6, 1 out of 50; Novocastra, Newcastle, UK); NK1 (NK1, 1 out of 75; Dako, Glostrup, Denmark)). Potential cytolytic cells were stained with Granzyme B (GrB-7, 1 out of 25; Dako, Glostrup, Denmark) and TiA1 (2G9, 1 out of 50; Immunotech, Marseille, France). TiA1 is a 17 kDa cytoplasmic granule-associated protein expressed in cells possessing, like Granzyme B-expressing cells, potential cytolytic activity. If residual tumour cells were identified, immunohistochemical analysis of HER2 expression was performed (A485, 1 out of 1600; Dako, Glostrup, Denmark).

Two pathologists, blinded to study treatment, performed independent IHC evaluations of all tumour samples. Infiltrating cells were analysed in four separate locations on each slide: *diffuse* (isolated cells distributed all around the area of tumour regression), *foci* (organised in nodules), *around* (a small rim around the tumour nodules in contact with residual tumour cells), and *inside* (penetrating inside tumour nodules between residual tumour cells). In each location on the slide, the number of stained cells was analysed using a semiquantitative ordinal scale ranging from 0 to 4 (0, +/−, ++, +++). For each antibody, this scale was established after the analysis of more than 15 cases and concerned only the number of infiltrating stained cells, whatever the intensity of the staining. The results of the analyses conducted by each independent pathologist were subsequently compared. For each location, all cases in which scores differed by more than one point on the semiquantitative scale were re-examined and a consensus score was reached.

### Statistical analysis

Slides were unblinded immediately prior to the statistical analysis. Statistical analyses were performed with a 5% type I (bilateral) error using the Stata 8.2 software package (StataCorp LP, TX, USA). The mean (standard deviation (s.d.)) number of stained cells was calculated for each data group (treatment group and two control groups). A two-sided Wilcoxon rank-sum (Mann–Whitney) test was used to determine differences between the groups.

A subgroup analysis was also performed to exclude potential treatment-independent, HER2 overexpression-related effects if values obtained for the treatment and control groups were significantly different. This analysis compared the results from the treatment group (TAXHER01) with those from HER2-overexpressing subgroups of the control groups (HER2 control, *n*=*15*). Due to multiple testing, a type I error adjustment was applied to this subgroup analysis (significance level *P*⩽0.01).

## RESULTS

Three patients from the TAXHER01 trial withdrew from the study due to toxicity. Seven additional cases were not available for immunohistochemical analysis because of fixative problems or false-positive HER2 overexpression. The final analysis was therefore performed on the surgical specimens from 23 patients. The control group from the GIREC01 trial comprised specimens obtained from 46 (23 NT–NT and 23 D) patients, 15 of whom had tumours that were HER2 positive (5 NT–NT and 10 D). Patient characteristic data are shown in Table 1.

### Pathologic response

After surgery, nine tumours in the TAXHER01 group (39%) were classified as demonstrating a pathological complete response (pCR; TA/NA or TA/NB), with two demonstrating residual tumour aggregates <2 mm in diameter. One other tumour was classified as TA/NC (no residual tumour in the breast, but residual tumour cells in the lymph nodes). The remaining 13 tumours displayed partial (pPR; TB or TC) or absent (TD) pathological responses. Eight tumours were classified as TB (residual tumour size 0.4–1.5 cm (mean 0.80 cm)), four were classified as TC (residual tumour size 1.5–7.0 cm (mean 3.2 cm)), and only one tumour was classified as TD (residual tumour size 6 cm). As tumour responses in the two control groups were matched to those in the treatment group, there were equivalent numbers of patients with complete, partial, or absent pathological responses in the 23 tumours treated in the D and NT–NT groups.

Areas of tumour response were associated with fibrosis, myxoedema, and macrophage/lymphocyte infiltration. Necrosis or infiltration with polynuclear neutrophils was not observed.

### Immunohistochemical analysis

#### Immune cell infiltration

Comparison of the three groups revealed that higher expression of PS100 and HLA-DR was observed in the TAXHER01 and NT–NT groups as compared with the D group, although the difference was not statistically significant (Table 2).

**Figure 1:**
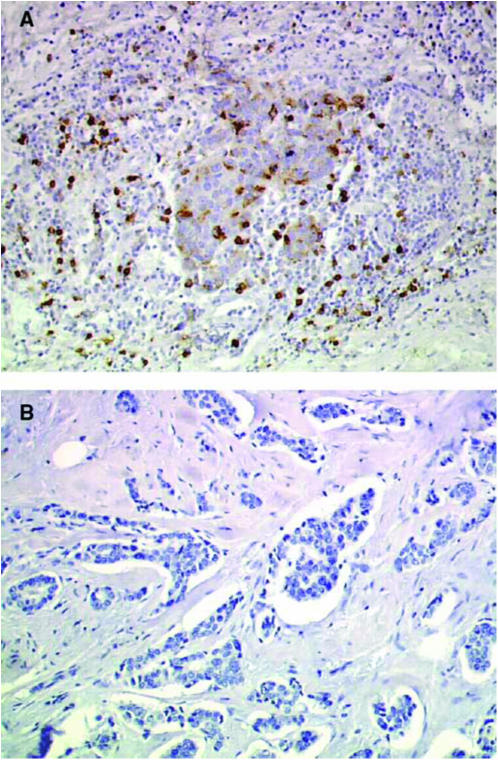
Immunohistochemical staining with NK1. Residual tumour from the TAXHER01 group (**A**) and from a matched tumour treated in the control group (**B**), both stained with NK1. The tumours of the TAXHER01 group show more cells in contact with or close to the tumour cells.

**Figure 2:**
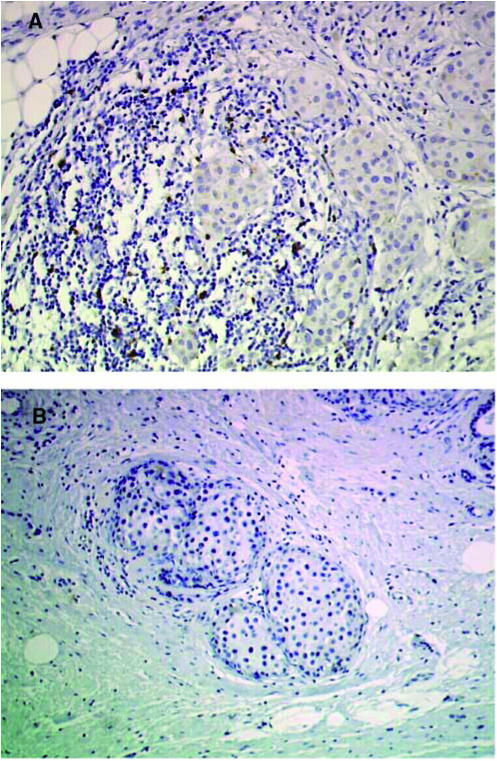
Immunohistochemical staining with CD56. Residual tumour from the TAXHER01 group (**A**) and from a matched tumour treated in the control group (**B**) both stained with CD56. The tumours of the TAXHER01 group show more cells in contact with or close to the tumour cells.

**Figure 3:**
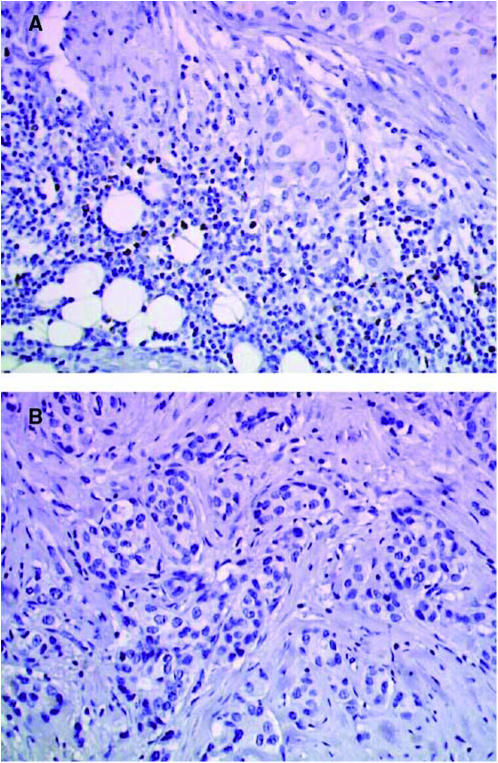
Immunohistochemical staining with Granzyme B. Residual tumour from the TAXHER01 group (**A**) and from a matched tumour treated in the control group (**B**), both stained with Granzyme B. The tumours of the TAXHER01 group show more cells in contact with or close to the tumour cells.

**Figure 4:**
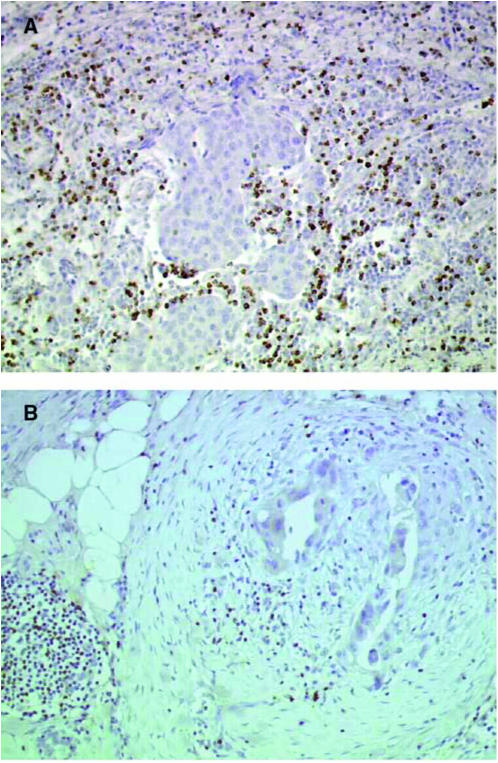
Immunohistochemical staining with TiA1. Residual tumour from the TAXHER01 group (**A**) and from a matched tumour treated in the control group (**B**), both stained with TiA1. The tumours of the TAXHER01 group show more cells in contact with or close to the tumour cells.

Comparison of pooled results from the D and NT–NT groups with TAXHER01 showed greater numbers of immunocompetent cells in the TAXHER01 specimens compared with controls. B lymphocytes were more numerous in the TAXHER01 specimens at the four locations analysed, but the difference was only significant in the *foci* infiltrates. T lymphocytes (CD3, CD4, and CD8) were also more numerous in the TAXHER01 specimens; differences between TAXHER01 and control specimens were statistically significant in the *diffuse* and *foci* infiltrates for CD3, in the *foci*, *inside* and *around* infiltrates for CD4, and in the *foci* and *around* infiltrates for CD8. Tumour area infiltration by macrophages (CD68) was not statistically different between the TAXHER01 and control groups. Dendritic cells (PS100, CD1a) and HLA-DR-expressing inflammatory cells appeared to be slightly more abundant in the TAXHER01 samples, but, because of the differences between the two control groups, results are difficult to interpret (Table 2). Figures 1, 2, 3 and 4 show immunohistochemical staining of cells that are able to undergo ADCC mechanisms, and that have potential cytotoxic activity, from both the TAXHER01 group and the case-matched control groups. Natural killer cell numbers were increased in the *around* (Figure 1A) and *inside* infiltrates of residual tumour cells in TAXHER01 specimens compared with controls (statistically significant by NK1 staining, Figure 1). In addition, there were statistically more NK1-positive cells in *foci* infiltrates from TAXHER01 specimens compared with controls. Staining with CD56 is notoriously difficult and was therefore less intense than other methods and consequently difficult to quantify (Figure 2). Finally, cytotoxic molecule (Granzymze B and TiA1)-expressing cells were more numerous in contact with the residual tumour cells in TAXHER01 specimens compared with controls (Figures 3 and 4), with statistically significant differences demonstrated for Granzyme B (around) and for TiA1 (inside).

#### Influence of HER2 overexpression on immune cell infiltration

To evaluate the potential treatment-independent effects of HER2 overexpression, immune cell infiltration in TAXHER01 tumours was compared with that in HER2-positive tumour subgroups of the control groups (HER2 control, *n*=*15*) (Table 3). While the number of cases in the HER2-positive tumour subgroup is low, the comparison between the TAXHER01 treatment group and this subgroup is favoured as these tumour samples were obtained from a clinical trial and hence had validated clinical and pathological data.

The limited number of cases in this subanalysis precludes definitive conclusions. There was a trend towards increased lymphocyte numbers (CD20, CD3, CD4, and CD8) in the TAXHER01 group compared with HER2 controls. Diffuse macrophage infiltration (CD68) was significantly increased in HER2 controls compared with the treatment group, but increased macrophage infiltration was observed around the residual tumours in the treatment group. There was also a trend towards increased dendritic cell (PS100, CD1a) and HLA-DR-expressing cell infiltration in TAXHER01 tumours. Natural killer and cytotoxic cells (TiA1 or Granzyme B) tended to be more numerous in contact with residual tumour cells in the TAXHER01 tumours, with significant differences demonstrated for TiA1 (*P*<0.05) and NK1 staining (*P*<0.01).

#### Correlation of tumour response with level of immune cell infiltration

To further characterise the role of immune cells on tumour regression, immune cell infiltration in TAXHER01 tumours demonstrating a partial response (TB) was compared with that in TAXHER01 tumours demonstrating a poor or absent response (TC and TD). For almost all markers (except CD8, CD68, and PS100), there was a trend towards increased cell infiltration in responsive tumours (Table 4). This was particularly evident for NK (CD56 and NK1) and cytotoxic markers (Granzyme B and TiA1) inside residual tumour aggregates. With the exception of TiA1, these differences were not statistically significant.

#### HER2 expression on residual tumour cells

At the time of surgery, HER2 overexpression was unaffected by trastuzumab treatment, with pre- and posttreatment biopsies demonstrating strong A485 staining on tumour cell membranes.

## DISCUSSION

Approximately 20–30% of all breast cancers overexpress HER2 (Slamon *et al*, 1987; van de Vijver *et al*, 1988; Ross *et al*, 2003; Owens *et al*, 2004). Targeted treatment of HER2-positive metastatic breast cancer (MBC) with single-agent trastuzumab demonstrates favourable efficacy (Cobleigh *et al*, 1999; Vogel *et al*, 2002), and efficacy is enhanced by combination with cytotoxic chemotherapy (Slamon *et al*, 2001; Marty *et al*, 2005). Indeed, combination treatment with trastuzumab and cytotoxic chemotherapy is now used as standard therapy for HER2-positive MBC. Trastuzumab is thought to have a diverse and complex mechanism of action. As trastuzumab is a monoclonal antibody that binds to the surfaces of HER2-overexpressing cancer cells, it has been postulated that ADCC may play an important role in the mechanism of action of this drug (Cooley *et al*, 1999; Clynes *et al*, 2000; Carson *et al*, 2001; Repka *et al*, 2003; Gennari *et al*, 2004). The current *in vivo* study was undertaken to analyse the potential role of different immune cells in the clinical response to trastuzumab.

Pre- and postoperative breast tissue samples were obtained from patients with HER2-overexpressing advanced breast cancer participating in a clinical trial of a neoadjuvant regimen incorporating trastuzumab and docetaxel. This treatment strategy is associated with a high pathologic response rate (Coudert *et al*, 2005), which is in accordance with the good efficacy previously reported for the combination of paclitaxel and trastuzumab in this setting (Bines *et al*, 2003; Burstein *et al*, 2003; Schiffhauer *et al*, 2003; Van Pelt *et al*, 2003; Baselga *et al*, 2004). In order to discount the effects of intratumoural immune cell modifications that may be induced by conventional chemotherapy drugs, the control group comprised tumours from patients treated with two different PST regimens (including and excluding docetaxel). Treatment and control groups were matched in respect of pathologic response to eliminate the effects of intratumoral immune cell modifications induced by the response itself. This is the first time a comparison of this type has been reported.

The results of this study demonstrate that the inclusion of trastuzumab in the PST regimen influences the number and topography of various immune cells, including T and B lymphocytes and NK cells, in tumour infiltrates. Natural killer cells are able to kill cells that are coated with an antibody via an ADCC mechanism (Clynes *et al*, 2000). The presence of increased numbers of NK cells in tumour infiltrates after trastuzumab treatment, as well as the presence of cytotoxic proteins such as Granzyme B, lends support to a role for NK cells in trastuzumab-induced tumour regression. Increased numbers of NK cells are also seen in tumours demonstrating an incomplete but pathologically important response (TB) compared with those showing a poor or absent pathologic response (TC or TD). The persistence of HER2 overexpression confirms that trastuzumab-mediated ADCC is a feasible mechanism of action for the drug.

Two *in vivo* pilot studies have evaluated the potential role of ADCC in the mechanism(s) of action of trastuzumab. Repka *et al* (2003) demonstrated that treatment with a combination of trastuzumab and interleukin-2 led to NK cell expansion and NK cell-mediated ADCC against HER2-overexpressing cells. Additionally, they showed that serum from treated patients had residual ADCC activity 2–8 weeks after the last trastuzumab injection. Gennari *et al* (2004) showed that peripheral blood mononuclear cells of trastuzumab-treated patients demonstrated *in vitro* cytopathic activity against HER2-overexpressing cells, with ADCC activity more pronounced in tumours demonstrating a good response to treatment compared with those exhibiting a poor response. In common with the present study, Gennari did not observe downregulation of HER2 expression after treatment, although they were able to demonstrate that residual tumour cells were still coated with trastuzumab at the time of the surgery as well as an increase in NK-rich lymphoid infiltration, but were unable to attribute this increase to the type of treatment or to the regression itself.

Further support for a role for ADCC in the mechanism of action of trastuzumab has emerged from *in vivo* xenograft (Clynes *et al*, 2000) and *in vitro* studies (Cooley *et al*, 1999; Carson *et al*, 2001; Kubo *et al*, 2003), which have demonstrated that NK cells are able to kill trastuzumab-coated HER2-overexpressing cells via a Fc*γ*RIII receptor (CD16)-mediated ADCC mechanism. Yamaguchi *et al* (2005) also demonstrated that lymphokine-activated killer cell cytotoxic activity against HER2-positive breast cancer cell lines MDA-MB453 and ZR75-1 was significantly increased in the presence of 10 nM trastuzumab. These data, together with the current data, lend support to the evaluation of combination therapy with trastuzumab and immunomodulatory agents, such as interleukin-2 (Fleming *et al*, 2002; Repka *et al*, 2003), as well as the construction of bispecific antibodies targeting HER2 and CD16 (Shahied *et al*, 2004).

Trastuzumab plus docetaxel is an approved and well-tolerated anticancer regimen that is used worldwide for the treatment of HER2-positive MBC. Taxanes, especially docetaxel, lead to increased serum concentrations of some cytokines and enhancement of NK cell activity (Tsavaris *et al*, 2002). The current study confirms that NK cytotoxicity via ADCC is probably one of the mechanisms of action of trastuzumab and demonstrates increased numbers of NK cells in tumours treated with docetaxel and trastuzumab compared with docetaxel alone. These findings may partially explain the synergistic activity of trastuzumab and docetaxel in the treatment of HER2-positive breast cancer and the excellent clinical outcomes afforded by this combination. In the TAXHER01 study (Coudert *et al*, 2005), tumours that were centrally confirmed to be HER2 positive displayed a pCR of 54%. This is superior to that observed with non-trastuzumab-containing neoadjuvant regimens in unselected patient populations. The results of this immunohistochemical study may also have implications for the design of future clinical trials involving trastuzumab. Perceived trastuzumab activity may be damaged by association with therapies that have immunosuppressive properties. An interesting area for future investigation could be combination therapy with trastuzumab, chemotherapy and immunomodulators, for example, co-administration of NK cells and/or cytokine injections.

## Figures and Tables

**Table 1:** Patient characteristics

	**TAXHER01 (*N*=23)**	**NT-NT (*N*=23)**	**D (*N*=23)**
Age, mean (range)	45 (24–64)	44.5 (27–60)	47.5 (27–65)
			
*Clinical tumour stage*			
T2	14	16	13
T3	9	7	10
			
*Clinical nodal status*			
N0	10	10	10
N1	13	13	13
			
*Scarff-Bloom-Richardson tumour grade*			
I	1	3	1
II	9	9	8
III	13	11	14
			
*Tumour hormone-receptor status*			
Positive	13	12	13
Negative	10	11	10

NT-NT=non-taxane, non-trastuzumab; D=docetaxel.

**Table 2:** Immune cell infiltration into breast tumours following PST

	**TAXHER01 (*n*=23)**	**Control (*n*=46)**	**D (*n*=23)**	**NT–NT (*n*=23)**	**TAXHER01 *vs* control**	**D *vs* NT–NT**
**Antibodies location**	**Mean (s.d.) staining intensity score**				***P*-value**	
*CD20*						
Diffuse	1.61 (1.3)	1.10 (0.9)	1.09 (0.9)	1.04 (0.9)	0.0802	0.8272
Around	1.13 (1.5)	0.52 (1.2)	0.39 (1.0)	0.65 (1.3)	0.0598	0.4432
Inside	0.43 (0.9)	0.33 (0.9)	0.04 (0.1)	0.61 (1.2)	0.4013	0.0686
Foci	3.13 (1.2)	1.70 (1.5)	1.61 (1.4)	1.78 (1.7)	**0.0005**	0.8391
						
*CD3*						
Diffuse	2.09 (1.4)	1.42 (1.0)	1.35 (0.9)	1.50 (1.1)	**0.0386**	0.7389
Around	1.52 (1.6)	0.82 (1.5)	0.70 (1.4)	0.95 (1.5)	0.0560	0.5014
Inside	0.65 (1.2)	0.47 (1.0)	0.39 (0.9)	0.55 (1.1)	0.4734	0.5014
Foci	2.87 (1.1)	1.64 (1.2)	1.43 (1.1)	1.86 (1.4)	**0.0001**	0.2116
						
*CD4*						
Diffuse	1.04 (1.3)	0.91 (0.9)	0.91 (0.8)	0.91 (0.9)	0.7605	0.8776
Around	1.35 (1.7)	0.46 (1.0)	0.39 (1.0)	0.52 (1.1)	**0.0270**	0.5223
Inside	0.35 (0.7)	0.04 (0.2)	0.00 (0.0)	0.09 (0.3)	**0.0209**	0.1527
Foci	2.17 (1.3)	1.30 (1.3)	1.65 (1.2)	0.96 (1.3)	**0.0107**	0.0627
						
*CD8*						
Diffuse	1.96 (1.5)	1.87 (0.9)	1.96 (0.9)	1.78 (1.0)	0.5670	0.4738
Around	1.56 (1.7)	0.76 (1.4)	0.52 (1.2)	1.00 (1.5)	**0.0460**	0.1997
Inside	1.22 (1.3)	0.78 (1.1)	0.65 (1.1)	0.91 (1.0)	0.1999	0.2454
Foci	2.39 (1.6)	1.50 (1.4)	1.43 (1.3)	1.56 (1.5)	**0.0160**	0.6410
						
*CD68*						
Diffuse	1.91 (1.3)	2.30 (1.2)	2.43 (1.2)	2.17 (1.2)	0.3036	0.5172
Around	0.91 (1.6)	0.54 (1.3)	0.48 (1.3)	0.61 (1.3)	0.3594	0.5271
Inside	0.56 (1.0)	0.39 (0.8)	0.26 (0.7)	0.52 (0.8)	0.5676	0.1883
Foci	0.09 (0.4)	0.00 (0.0)	0.00 (0.0)	0.00 (0.0)	0.1573	
						
*PS100*						
Diffuse	1.70 (1.3)	1.59 (1.2)	1.48 (0.9)	1.70 (1.5)	0.7068	0.9540
Around	1.35 (1.6)	0.63 (1.3)	0.30 (0.9)	0.96 (1.5)	**0.0557**	0.1234
Inside	0.61 (1.3)	0.37 (0.9)	0.13 (0.6)	0.61 (1.1)	0.4579	**0.0488**
Foci	1.74 (1.7)	1.1 (1.4)	0.78 (1.2)	1.35 (1.6)	**0.0691**	0.2202
						
*CD1a*						
Diffuse	1.61 (0.8)	1.83 (0.9)	1.74 (0.8)	1.91 (0.9)	0.3788	0.4385
Around	0.39 (0.7)	0.41 (1.0)	0.39 (1.0)	0.43 (1.0)	0.5556	0.5378
Inside	0.26 (0.5)	0.28 (0.9)	0.17 (0.7)	0.39 (1.0)	0.3180	0.5968
Foci	0.00 (0.0)	0.00 (0.0)	0.00 (0.0)	0.00 (0.0)		
						
*HLA-DR*						
Diffuse	2.09 (1.0)	2.33 (1.0)	2.26 (0.8)	2.39 (1.1)	0.5370	0.4624
Around	1.70 (1.7)	1.04 (1.5)	0.83 (1.5)	1.26 (1.5)	0.1151	0.2308
Inside	0.91 (1.2)	0.72 (1.0)	0.39 (0.8)	1.04 (1.1)	0.5778	**0.0258**
Foci	2.30 (1.1)	1.54 (1.3)	1.70 (1.3)	1.39 (1.3)	**0.0335**	0.4649
						
*CD56* a						
Diffuse	0.83 (1.0)	0.74 (0.8)	0.87 (0.8)	0.61 (0.8)	1.0000	0.2125
Around	1.00 (1.5)	0.57 (1.1)	0.43 (1.0)	0.70 (1.2)	0.2468^a^	0.3175
Inside	0.30 (0.7)	0.11 (0.4)	0.09 (0.4)	0.13 (0.3)	0.2520^a^	0.3336
Foci	0.26 (0.7)	0.28 (0.8)	0.26 (0.9)	0.30 (0.7)	0.9739	0.4756
						
*NK1*						
Diffuse	1.45 (1.4)	1.20 (0.9)	1.39 (0.8)	1.00 (0.9)	0.5864	0.1349
Around	1.64 (1.6)	0.41 (1.0)	0.43 (1.1)	0.39 (1.0)	**0.0003**	0.7919
Inside	0.82 (1.3)	0.30 (0.8)	0.17 (0.7)	0.43 (1.0)	**0.0438**	0.2251
Foci	2.01 (1.4)	0.74 (1.1)	0.83 (1.1)	0.65 (1.1)	**0.0003**	0.4678
						
*TiA1*						
Diffuse	1.13 (1.3)	1.30 (1.0)	1.30 (0.9)	1.30 (1.1)	0.4411	0.9816
Around	1.43 (1.7)	0.85 (1.5)	0.61 (1.4)	1.1 (1.6)	0.1982	0.3113
Inside	0.65 (1.0)	0.26 (0.8)	0.17 (0.7)	0.35 (0.9)	**0.0370**	0.3878
Foci	1.00 (1.0)	0.54 (1.0)	0.65 (1.2)	0.43 (0.8)	0.2181	0.5759
						
*Granzyme B*						
Diffuse	0.26 (0.7)	0.26 (0.4)	0.30 (0.5)	0.22 (0.4)	0.4870	0.5066
Around	0.83 (1.3)	0.28 (0.9)	0.22 (0.9)	0.35 (0.9)	**0.0324**	0.4087
Inside	0.22 (0.7)	0.07 (0.3)	0.13 (0.5)	0.00 (0.0)	0.1926	0.1528
Foci	0.52 (0.8)	0.33 (0.7)	0.13 (0.3)	0.52 (0.9)	0.2571	0.1126

NT–NT, anthracycline-based PST that does not include a taxane or trastuzumab; D, docetaxel-based PST.

For diffuse/around/inside/foci, scores range from 0 to 4.

a Staining with CD56 was less intense and difficult to quantify.

Significant if *P*<0.05. Bold values signify their importance in the results and the discussion.

**Table 3:** Immune cell infiltration into HER2-overexpressing breast tumours following PST

	**TAXHER01 (*n*=23)**	**HER2 control (*n*=15)**	**TAXHER01 *vs* control**
**Antibodies location**	**Mean (s.d.) staining intensity score**		***P*-value**
*CD20*			
Diffuse	1.61 (1.27)	1.53 (1.19)	0.8525
Around	1.13 (1.52)	0.40 (1.06)	0.1036
Inside	0.43 (0.94)	0.20 (0.77)	0.2488
Foci	3.13 (1.18)	2.13 (1.64)	0.0840
			
*CD3*			
Diffuse	2.09 (1.41)	1.80 (1.08)	0.3734
Around	1.52 (1.62)	0.93 (1.44)	0.2352
Inside	0.65 (1.19)	0.40 (0.74)	0.6948
Foci	2.87 (1.06)	1.67 (1.40)	**0.0046**
			
*CD4*			
Diffuse	1.04 (1.33)	0.87 (0.91)	0.9618
Around	1.35 (1.67)	0.33 (0.72)	0.0605
Inside	0.35 (0.71)	0.00 (0.00)	0.0565
Foci	2.17 (1.27)	1.53 (1.30)	0.1423
			
*CD8*			
Diffuse	1.96 (1.49)	2.00 (1.00)	0.9259
Around	1.56 (1.70)	0.80 (1.21)	0.1342
Inside	1.22 (1.31)	0.67 (0.90)	0.2039
Foci	2.39 (1.62)	2.33 (1.17)	0.5882
			
*CD68*			
Diffuse	1.91 (1.31)	3.13 (0.74)	**0.0037**
Around	0.91 (1.59)	0.00 (0.00)	0.0341
Inside	0.56 (0.99)	0.33 (0.72)	0.5592
Foci	0.09 (0.42)	0.00 (0.00)	0.4193
			
*PS100*			
Diffuse	1.70 (1.29)	2.00 (1.07)	0.4600
Around	1.35 (1.64)	0.67 (1.29)	0.2253
Inside	0.61 (1.31)	0.20 (1.56)	0.4394
Foci	1.74 (1.66)	1.27 (1.53)	0.3246
			
*CD1a*			
Diffuse	1.61 (0.78)	1.80 (0.77)	0.4990
Around	0.39 (0.72)	0.40 (0.91)	0.7634
Inside	0.26 (0.54)	0.27 (0.80)	0.5800
Foci	0.00 (0.00)	0.00 (0.00)	
			
*HLA-DR*			
Diffuse	2.09 (1.04)	2.40 (0.91)	0.5216
Around	1.69 (1.72)	0.73 (1.33)	0.0786
Inside	0.91 (1.16)	0.53 (0.92)	0.3676
Foci	2.30 (1.15)	2.00 (1.20)	0.5809
			
*CD56* a			
Diffuse	0.83 (1.03)	0.67 (0.82)	0.7562
Around	1.00 (1.51)	0.80 (1.08)	0.8762
Inside	0.30 (0.70)	0.20 (0.56)	0.7062
Foci	0.26 (0.69)	0.53 (1.12)	0.4797
			
*NK1*			
Diffuse	1.45 (1.40)	1.20 (0.86)	0.5737
Around	1.64 (1.56)	0.40 (1.12)	**0.0097**
Inside	0.82 (1.26)	0.20 (0.77)	**0.0532**
Foci	2.05 (1.43)	1.07 (1.16)	**0.0303**
			
*TiA1*			
Diffuse	1.13 (1.32)	1.67 (0.82)	0.1448
Around	1.43 (1.73)	0.53 (1.41)	0.0942
Inside	0.65 (1.03)	0.07 (0.26)	**0.0407**
Foci	1.00 (1.20)	1.00 (1.51)	0.8120
			
*Granzyme B*			
Diffuse	0.26 (0.69)	0.33 (0.49)	0.3126
Around	0.82 (1.33)	0.27 (0.80)	0.1374
Inside	0.22 (0.67)	0.07 (0.26)	0.5188
Foci	0.52 (0.85)	0.27 (0.80)	0.1758

NT–NT, anthracycline-based PST that does not include a taxane or trastuzumab; D, docetaxel-based PST.

For diffuse/around/inside/foci, scores range from 0 to 4.

a Staining with CD56 was less intense and difficult to quantify. Significant if *P*<0.05. Bold values signify their importance in the results and the discussion.

**Table 4:** Immune cell infiltration into breast tumours following trastuzumab-based PST by response status

	**TAXHER01 TB (*n*=8)**	**TAXHER01 TC/TD (*n*=5)**	
**Antibodies location**	**Mean (s.d.) staining intensity score**		***P*-value**
*CD20*			
Diffuse	1.50 (1.51)	1.40 (1.14)	1.0000
Around	2.75 (1.28)	0.8 (1.10)	**0.0187**
Inside	1.13 (1.36)	0.20 (0.45)	0.2090
Foci	3.63 (0.52)	3.40 (0.89)	0.7339
			
*CD3*			
Diffuse	1.50 (1.41)	1.60 (1.52)	0.9381
Around	3.25 (0.71)	1.80 (1.30)	**0.0362**
Inside	1.75 (1.49)	0.20 (0.45)	**0.0437**
Foci	3.00 (0.76)	3.20 (0.45)	0.6134
			
*CD4*			
Diffuse	0.88 (1.64)	0.60 (1.34)	0.7659
Around	2.75 (1.49)	1.80 (1.64)	0.1852
Inside	0.75 (1.04)	0.40 (0.55)	0.6737
Foci	2.50 (1.31)	2.80 (0.84)	0.9364
			
*CD8*			
Diffuse	2.13 (1.36)	0.80 (1.79)	0.2037
Around	2.88 (1.25)	2.60 (1.52)	0.7362
Inside	1.75 (1.04)	2.80 (0.45)	**0.0503**
Foci	2.38 (1.69)	2.00 (1.87)	0.7045
			
*CD68*			
Diffuse	0.88 (1.25)	2.40 (1.34)	**0.0467**
Around	2.13 (1.81)	0.80 (1.79)	0.2590
Inside	0.88 (1.25)	1.20 (1.10)	0.6215
Foci	0.25 (0.71)	0.00 (0.00)	0.4292
			
*PS100*			
Diffuse	1.50 (1.51)	2.20 (1.30)	0.3635
Around	2.25 (1.58)	2.60 (1.52)	0.6471
Inside	1.50 (1.85)	0.40 (0.89)	0.2418
Foci	1.75 (1.91)	2.60 (1.52)	0.5323
			
*CD1a*			
Diffuse	1.50 (0.93)	1.20 (0.45)	0.4666
Around	0.88 (0.83)	0.40 (0.89)	0.2590
Inside	0.63 (0.74)	0.20 (0.45)	0.2693
Foci	0.00 (0.00)	0.00 (0.00)	
			
*HLA-DR*			
Diffuse	2.00 (1.31)	2.00 (1.22)	0.8750
Around	3.13 (1.36)	2.80 (0.45)	0.1454
Inside	1.75 (1.04)	1.40 (1.34)	0.6471
Foci	2.50 (1.31)	2.60 (0.55)	1.0000
			
*CD56* a			
Diffuse	0.75 (1.16)	0.40 (0.89)	0.5306
Around	2.25 (1.67)	1.00 (1.41)	0.1460
Inside	0.88 (0.99)	0.00 (0.00)	0.0714
Foci	0.00 (0.00)	0.40 (0.89)	0.2059
			
*NK1*			
Diffuse	0.57 (1.13)	1.00 (1.41)	0.6282
Around	3.00 (1.41)	2.20 (0.84)	0.1063
Inside	1.29 (1.50)	1.20 (1.30)	1.0000
Foci	2.71 (1.38)	2.00 (1.22)	0.2332
			
*TiA1*			
Diffuse	0.63 (1.19)	1.00 (1.41)	0.5902
Around	3.13 (1.36)	1.60 (1.52)	**0.0456**
Inside	1.50 (1.20)	0.60 (0.89)	0.1697
Foci	0.50 (1.41)	0.60 (1.34)	0.8160
			
*Granzyme B*			
Diffuse	0.50 (1.07)	0.20 (0.45)	0.7659
Around	2.00 (1.60)	0.60 (0.89)	0.1107
Inside	0.63 (1.06)	0.00 (0.00)	0.1366
Foci	0.25 (0.71)	0.80 (1.30)	0.2762

For diffuse/around/inside/foci, scores range from 0 to 4.

a Staining with CD56 was less intense and difficult to quantify.

Significant if *P*<0.01 (multiple testing adjustment). Bold values signify their importance in the results and the discussion.
